# Frontal midline theta rhythm and gamma power changes during focused attention on mental calculation: an MEG beamformer analysis

**DOI:** 10.3389/fnhum.2014.00406

**Published:** 2014-06-11

**Authors:** Ryouhei Ishii, Leonides Canuet, Tsutomu Ishihara, Yasunori Aoki, Shunichiro Ikeda, Masahiro Hata, Themistoklis Katsimichas, Atsuko Gunji, Hidetoshi Takahashi, Takayuki Nakahachi, Masao Iwase, Masatoshi Takeda

**Affiliations:** ^1^Department of Psychiatry, Osaka University Graduate School of MedicineSuita, Japan; ^2^Department of Cognitive and Computational Neuroscience, Centre for Biomedical Technology, Complutense University of Madrid, UPMMadrid, Spain; ^3^Osaka Psychiatric Medical CenterHirakata, Japan; ^4^Faculty of Education and Human Sciences, Course of School Education, Yokohama National UniversityYokohama, Japan; ^5^Department of Child and Adolescent Mental Health, National Center for Neurology and Psychiatry, National Institute of Mental HealthKodaira, Japan

**Keywords:** frontal midline theta, focused attention, arithmetic calculation, gamma band, magnetoencephalography (MEG), synthetic aperture magnetometry (SAM), beamformer, spatial filtering

## Abstract

Frontal midline theta rhythm (Fmθ) appears widely distributed over medial prefrontal areas in EEG recordings, indicating focused attention. Although mental calculation is often used as an attention-demanding task, little has been reported on calculation-related activation in Fmθ experiments. In this study we used spatially filtered MEG and permutation analysis to precisely localize cortical generators of the magnetic counterpart of Fmθ, as well as other sources of oscillatory activity associated with mental calculation processing (i.e., arithmetic subtraction). Our results confirmed and extended earlier EEG/MEG studies indicating that Fmθ during mental calculation is generated in the dorsal anterior cingulate and adjacent medial prefrontal cortex. Mental subtraction was also associated with gamma event-related synchronization, as an index of activation, in right parietal regions subserving basic numerical processing and number-based spatial attention. Gamma event-related desynchronization appeared in the right lateral prefrontal cortex, likely representing a mechanism to interrupt neural activity that can interfere with the ongoing cognitive task.

## Introduction

Frontal midline theta rhythm (Fmθ) is a distinct train of focal 5–7 Hz theta waves which appears over medial frontal areas in the EEG of normal subjects when performing a broad range of cognitive tasks demanding mental concentration (Ishihara and Yoshi, 1972; Iramina et al., 1996; Sasaki et al., 1996; Ishii et al., 1999). Thus, this brain activity is considered to reflect focused attentional processing. Reports of enhanced Fmθ in the pre-shot phase of rifle shooting (Doppelmayr et al., 2008), during car driving (Laukka et al., 1995) and in meditation states (Aftanas and Golocheikine, 2001) provide support to this notion.

Since the first report of Fmθ made by Ishihara and Yoshi (1972), Fmθ has been investigated in a number of neurophysiological and neuroimaging studies. Earlier studies of Fmθ using scalp EEG reported widespread distribution of this activity in midfrontal sites, but an accurate identification of its cortical generators within the medial frontal cortex was lacking (Mizuki et al., 1980; Laukka et al., 1995; Iramina et al., 1996). This is mainly due to the low spatial resolution of EEG. To overcome this problem, a few studies looking at the anatomical correlates of Fmθ used fMRI with simultaneous EEG recording. Despite the fact that fMRI scanner noise may affect mental concentration in some individuals when engaged in mental reasoning tasks (Pripfl et al., 2006), these fMRI studies clearly visualized Fmθ activity localized to the anteromedial frontal cortex (Gevins et al., 1997; Mizuhara et al., 2004; Sammer et al., 2007).

In an attempt to visualize the magnetic counterpart of the EEG-recorded Fmθ activity and clarify its cortical sources, we previously used MEG in four normal subjects and found that a large area over the bilateral medial prefrontal cortex generated Fmθ during continuous mental calculation (Ishii et al., 1999). Thus, our findings provided further support for a specific role of the prefrontal cortex in focused attentional processing. Findings from another MEG study using different types of attention-demanding tasks, including mental calculation, suggested that Fmθ reflects alternative activation of the prefrontal and anterior cingulate cortex (ACC) in the human brain (Asada et al., 1999). Overall, neuroimaging MEG research taking advantage of the excellent temporal resolution and higher spatial resolution of this technique compared to EEG, has shed some light on the generators of Fmθ, and supported the concept that Fmθ reflects activation of neural networks involved in allocation of attention related to various types of cognitive stimuli.

To generate Fmθ and understand the neural correlates of attentional processing, arithmetic calculation has often been used as the attention demanding task (Mizuki et al., 1980; Iramina et al., 1996; Sasaki et al., 1996; Asada et al., 1999; Ishii et al., 1999). However, apart from attention-induced neural activity, little has been reported on activation patterns related to calculation itself in Fmθ experiments. Indeed, there have been few attempts to identify whether source-power changes in frequency bands other than theta also emerge when focusing attention on mental calculation. Recent neurophysiological studies looking at EEG event-related responses in mental addition and subtraction using a calculation strategy approach have focused on theta and alpha oscillatory activity (De Smedt et al., 2009; Grabner and De Smedt, 2011). Evidence from neuropsychological and neuroimaging studies indicate that several cortical areas across hemispheres are implicated in arithmetic processing (Menon et al., 2000; Gruber et al., 2001; Dehaene et al., 2003, 2004; Kong et al., 2005; Fehr et al., 2007; Ischebeck et al., 2009). For instance, multiplication operations, which require retrieval of arithmetic facts stored in rote verbal memory (verbal number manipulation) mainly induce activation of the left angular gyrus (Gruber et al., 2001; Dehaene et al., 2003; Ischebeck et al., 2009). This area is also implicated in complex arithmetic operations (Menon et al., 2000; Dehaene et al., 2003; Grabner et al., 2007) along with other regions such as the left inferior temporal gyrus (Gruber et al., 2001; Kong et al., 2005) and the inferior and medial parietal cortex (Chochon et al., 1999; Kong et al., 2005). In contrast, addition and subtraction, which require genuine numerical calculation (quantity representations), have often been reported associated with activation of the parietal cortex (Chochon et al., 1999; Menon et al., 2000; Dehaene et al., 2003, 2004; Fehr et al., 2007). This indicates that the large variation in cortical sources of arithmetic-induced activation across studies may be due to the existence of sharp disassociations between arithmetic operations (i.e., addition, subtraction, multiplication, and division) and calculation complexity.

Although substantial progress has been made toward characterizing the anatomical correlates of arithmetic operations, the underlying neural activity (i.e., oscillations in different frequency bands) has been largely unexplored. Findings from EEG studies on arithmetic processing suggested that engagement in simple mental calculation may be associated particularly with oscillatory activity power changes in the gamma band (Micheloyannis et al., 2002). There is increasing evidence that gamma oscillations are also involved in a variety of cognitive processes including visuospatial focused attention (Kaiser and Lutzenberger, 2003), visual perception, learning and memory (Kaiser and Lutzenberger, 2005). However, little has been reported on the possible implication of gamma oscillations in calculation-related attention or in arithmetic operations. In fact, previous MEG investigations based on mental calculation paradigms often used single dipole analysis to localize specifically theta activity sources (Iramina et al., 1996; Sasaki et al., 1996; Asada et al., 1999). Because cognitive processing is functionally related to serial and parallel activation of multiple brain regions (Ishii et al., 2009) as well as to cortical oscillations in different frequency bands (Pfurtscheller and Lopes da Silva, 1999), applying MEG-dipole models which identify center of gravity rather than the volume of activation (Herdman et al., 2003), and focusing exclusively in theta oscillations, might not be sufficient to visualize the extended network of sources related to focused attention and mental calculation in the human brain. Hence, the application of methods which can detect cognitive task-induced oscillatory response and localize the underlying cortical sources may help elucidate the role of cortical oscillations in mental arithmetic processing.

Considerable insight into the dynamics of oscillatory activity across the cortex is provided by beamformer, owing to its action as a spatially selective filter to MEG signals. This allows estimation of the oscillatory activity coming from a given location in the brain (Hillebrand et al., 2005). Furthermore, by applying beamformer in both the active and control time windows (e.g., during and prior to stimulation), task-related power changes in brain electric or magnetic activity can be assessed, as well (Vrba and Robinson, 2001; Brookes et al., 2007). Synthetic aperture magnetometry (SAM), a spatially filtering technique based on non-linear constrained minimum-variance beamformer, permits unambiguous three-dimensional mapping of cortical power changes within specific frequency bands during task performance. The accuracy of this map, however, relies on the correctness of the beamformer assumptions for the given data set (Robinson and Vrba, 1998; Ishii et al., 1999, 2009; Hillebrand et al., 2005). Using this method along with permutation tests for statistical group analysis of MEG data, we could accurately identify neural sources and underlying oscillatory activity power changes that were functionally engaged in auditory attention and memory updating process (Ishii et al., 2009), cortical organization of sensorimotor areas (Ishii et al., 2002), singing and vocalization (Gunji et al., 2007), and perceptual information processing (Herdman et al., 2003; Doesburg et al., 2013). Thus, SAM beamformer and permutation analysis have proven to be useful methods to visualize sources of cognitive task-induced oscillatory activity in the brain.

The purpose of this study was to use spatially filtered MEG by SAM technique, and permutation analysis to precisely localize cortical generators of the magnetic counterpart of Fmθ as well as other sources of oscillatory activity associated with mental calculation, particularly with arithmetic subtraction, as it requires genuine numerical calculation or quantity representation.

## Methods

### Subjects

Eleven healthy volunteers, who were mainly researchers at Osaka university (six males, aged 27–36 years, mean age 32 years), participated in this study. The subjects had no specific education or background that could facilitate mental calculation. All subjects were right-handed, as assessed by the Edinburgh Handedness Inventory (Oldfield, 1971). Informed consent was obtained from all subjects prior to the experiments. The study was performed in accordance with the Declaration of Helsinki, and approved by the Ethics Committee of the Osaka University Hospital.

### Experimental design

**Figure 1:**
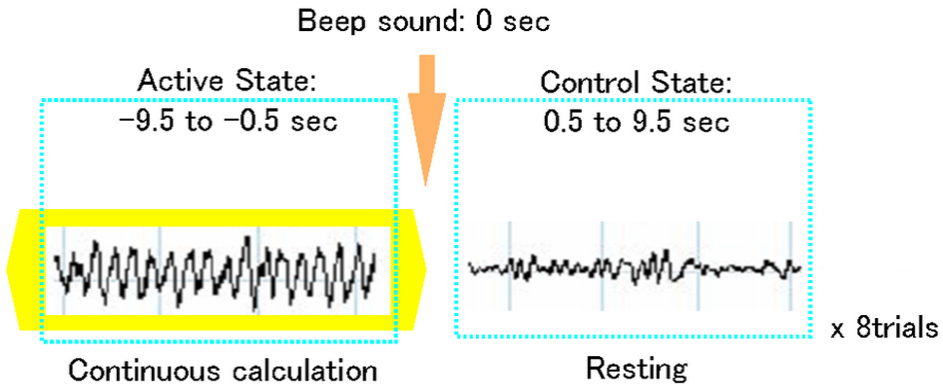
**Schematic representation of an MEG trial using a focused attention paradigm**. Each trial included a 10-s frontal midline theta activity during focused attention on mental calculation (active state) followed by a 10-s non-arithmetic period (control state).

The experiment consisted of two conditions: (1) eye-closed resting state (control interval) and (2) mental arithmetic state (active interval). During the mental arithmetic state, the subjects were asked to serially subtract 7 from 1000, as fast as possible, with their eyes closed, thereby generating Fmθ. This paradigm is also assumed to elicit activation of cortical areas subserving genuine numerical calculation or quantity manipulation, as subtraction problems involve more quantity representation compared to division operations, multiplication tables and small exact addition facts, that can be stored in rote verbal memory (Menon et al., 2000; Dehaene et al., 2003). When enhanced, rhythmic theta oscillations lasting for at least 10 s. were visually identified in the MEG recordings, a beeping sound was given to indicate the end of the arithmetic state and the beginning of the resting state for 10 s. (Figure 1). Thus, resting and mental calculation states were alternately recorded on each subject for a total of 8 trials, each one of 20-s duration. The task was carried out purely mentally to avoid movement-related artifacts. Subject's performance of mental arithmetic was not controlled during the MEG recording. However, prior to the experiments, like in previous functional neuroimaging studies using covert mental arithmetic (Rueckert et al., 1996; Kawashima et al., 2004; Mizuhara et al., 2004), practice sessions (serial subtractions) were performed. In these sessions, the subjects provided answers orally, and the accuracy of the calculations was checked. This served as an index of each subjects' arithmetic proficiency. All study participants showed an excellent performance in the practice trials.

### MEG data acquisition

MEG recordings were performed on all subjects in a magnetically shielded room using a helmet-shaped whole-head array of 64-channel SQUID sensor (NeuroSQUID Model 100, CTF Systems Inc.). Each of the 64 primary sensors used a first-order axial gradiometer flux transformer. Ambient magnetic noise was reduced further by synthesizing third-order gradiometer response in firmware using the reference SQUID sensor array (Vrba and Robinson, 2001). This was especially effective at reducing low-frequency noise. The data were recorded with the subject sitting on a comfortable chair with the head positioned in the helmet-shaped Dewar. A head position indicator with three small coils, placed at the nasion and bilateral preauricular points, was fixed on the scalp. MEG signals were digitized at a sample rate of 250 Hz, and filtered using a 60 Hz notch filter and 100 Hz low pass filter. The resulting data were recorded on disk and analyzed offline.

### Anatomical MRI

To convert the sources of MEG oscillatory activities into subjects' brain images, magnetic resonance imaging (MRI) scans were obtained for all subjects using a 1.0-T MRI system (Magneton Impact, SIEMENS Inc., Germany) or a 1.5-T Siemens Magnetom Vision plus system (Siemens, Erlangen, Germany). MRI data consisted of T1-weighted axial anatomical images with an in-plane resolution of 256 × 192 and 124 sagittal slices (1.4 mm thickness). Anatomical landmarks (i.e., nasion and bilateral preauricular points) were used to create an MEG head-based three-dimensional coordinate system.

### Time frequency analysis

Brain Electrical Source Analysis (BESA 5.0, MEGIS Software GmbH, Grafelfing, Germany) software was used to visualize time-frequency representations for MEG sensors in each subject. The BESA beamformer applies complex demodulation to transform time-domain MEG data into time-frequency data (Hoechstetter et al., 2004). This provides information on the envelope amplitude and the phase of a specified frequency band as a function of time. The complex demodulation consisted of a multiplication of the time-domain signal by a complex periodic potential function with a frequency equal to the frequency analyzed, followed by a low-pass finite impulse response (FIR) filter of Gaussian shape. This is equivalent to a wavelet transformation with constant wavelet width across frequencies. In the resulting complex signal, its magnitude corresponds to half the envelope amplitude of the filtered frequency band and its phase to the compound phase at that frequency. We analyzed the frequency range of 4–60 Hz in 2-Hz steps with a time sampling rate of 25 ms-steps. To obtain power values, the time-series MEG data were squared and averaged across all single trials under the respective conditions. From this time-frequency transformation, event-related synchronization (ERS) and event-related desynchronization (ERD) measures are obtained. ERS was denoted as an increase in power of oscillatory activity in a given frequency band during the mental arithmetic state (active interval) relative to the mean power during the resting state (control interval). The opposite phenomenon, suppression of rhythmic brain activity during the mental task compared to a rebound after the task, was denoted as ERD.

### Within-subject source localization analysis (SAM analysis)

The spatial distributions of ERS or ERD in different frequency bands functionally related to focused attention on mental calculation were estimated from the unaveraged MEG measurements using Synthetic Aperture Magnetometry (SAM) analysis (Robinson and Vrba, 1998; Ishii et al., 1999, 2009). The data were first subjected to the following bandpass filters: 4–8 Hz (theta), 8–15 Hz (alpha), 15–30 Hz (beta), and 30–60 Hz (gamma). Then, SAM was used to generate a 16 × 12 × 12 cm volumetric image of root-mean squared (RMS) source activity from the filtered MEG signals, with a 2.5 mm voxel resolution. As an adaptive beamformer, SAM applies a spatial filter specific for each brain voxel, to suppress the interference of unwanted signals from other locations including the environmental noise, thus estimating source power with high spatial resolution (Robinson and Vrba, 1998). The spatial filter at a given location is a linear projection operator defined by a set of coefficients, with one coefficient for each sensor, which is determined by minimizing the source power under a constraint of unity gain at the location of interest. Finally, the Student's t statistic was computed, on a voxel-by-voxel basis, as the difference between the estimated source power for the active (8 epochs of 10-s Fmθ during mental calculation) and the control (8 epochs of 10-s non-Fmθ during resting following mental calculation) states, divided by their ensemble standard error that included both instrumental (SQUID sensor) noise and experimental variance. The resulting functional image represents a Student's t statistic parametric map, which was then fused with the corresponding MRI, relating brain anatomy to function. The optimum orientation for the spatial filter at location of interest is determined by rotation of the source orientation in the tangential plane; the orientation at which the pseudo-Z statistic is maximized is then used as the optimum orientation for the source strength estimate at each location. Details on SAM procedures are described elsewhere (Robinson and Vrba, 1998; Ishii et al., 2009).

### Statistic group analysis (permutation tests)

For statistical analyses of the group data, the distribution of each individual's SAM image was transformed into a common anatomical space, the SPM T1 template space (Barnes and Hillebrand, 2003). First, the SAM volumes of each subject were co-registered with his/her three-dimensional anatomical MRI based on fiducial positions measured during the MEG acquisition. Transformation parameters that map the subject's MRI to the template space were then determined using SPM99 software (Wellcome Department of Cognitive Neurology, London, UK). The spatial normalized subject's data were subsequently obtained by applying the above transformation to the SAM volumes. A non-parametric permutation technique was applied to the normalized SAM results (SAM-permutation statistics) to determine voxels with significant values by comparing the grand mean pseudo *t*-value of a voxel and the distribution of permuted pseudo *t*-values. This distribution was computed by randomly rearranging the active and control conditions and averaging the newly calculated pseudo *t*-values. The omnibus null hypothesis of “no activation” anywhere in the brain was rejected if at least one *t*-value was above the critical threshold for α < 0.05 determined by 1024 permutations, thus correcting for multiple testing. Voxels with pseudo *t*-values above this critical 0.05 threshold were deemed regions of activation, and the corresponding voxels were then overlaid on a normalized structural MRI. For details of this procedure see a study by Chau et al. (2004) and our previous report (Ishii et al., 2013).

## Results

Focusing attention on mental calculation, specifically on serial arithmetic subtraction, resulted in significant source-power changes in theta and gamma frequency bands over different cortical regions. There were no significant power changes in any of the other frequency bands. Table 1 summarizes the cortical distribution of task-related activation, as indicated by SAM-permutation analysis.

**Table 1:** **Cortical regions showing significant task-related activation or deactivation in different frequency bands**.

**Cortical regions**	**BAs**	**Cluster size**	**Talairach coordinates**			**Pseudo *t*-value**
			***x***	***y***	***z***	
*Theta ERS*		148				
Right medial prefrontal	9		3	37	33	1.62
Left medial prefrontal	8		−10	47	43	1.50
Right anterior cingulate	32		4	30	29	1.47
*Gamma ERS*						
Right posterior parietal	40	31	50	39	57	1.92
*Gamma ERD*						
Right inferior frontal	44	58	50	14	15	−1.63

*ERS, Event-related synchronization; ERD, Event-related desynchronization; BAs, Brodmann areas*.

### Theta power changes: frontal midline theta (Fmθ) activity

**Figure 2:**
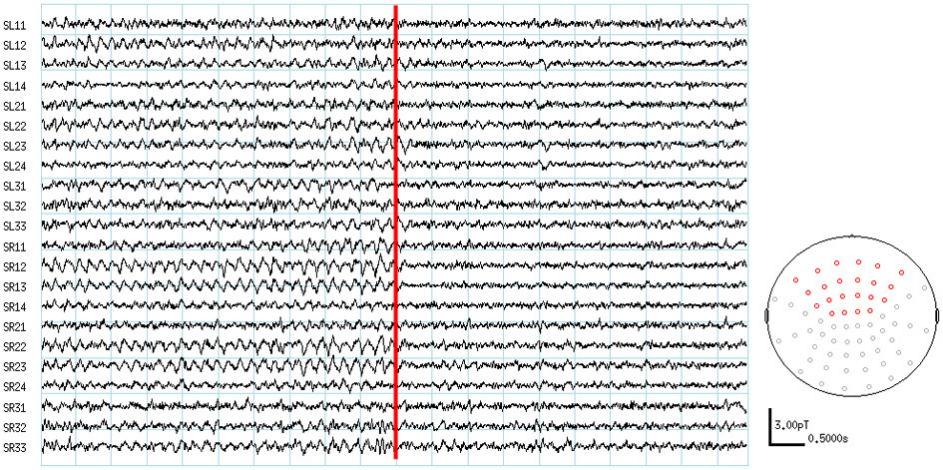
**MEG waveforms during the active and control conditions**. The location of channels showing frontal theta enhancement are indicated in red color on the MEG sensor map.

**Figure 3:**
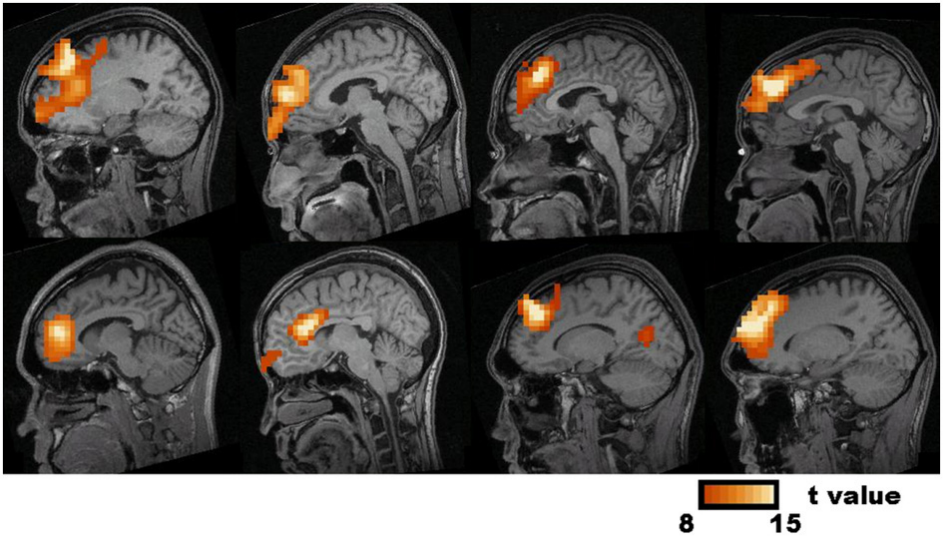
**Synthetic Aperture Magnetometry analysis of eight subjects who had prominent medial frontal source power changes in theta frequency band in individual analysis**. Statistic parametric maps (SPM) of the Frontal Midline Theta source-current density in individual subjects are projected onto sagittal slices of the subject's MRI. The color bar represents *t*-values.

**Figure 4:**
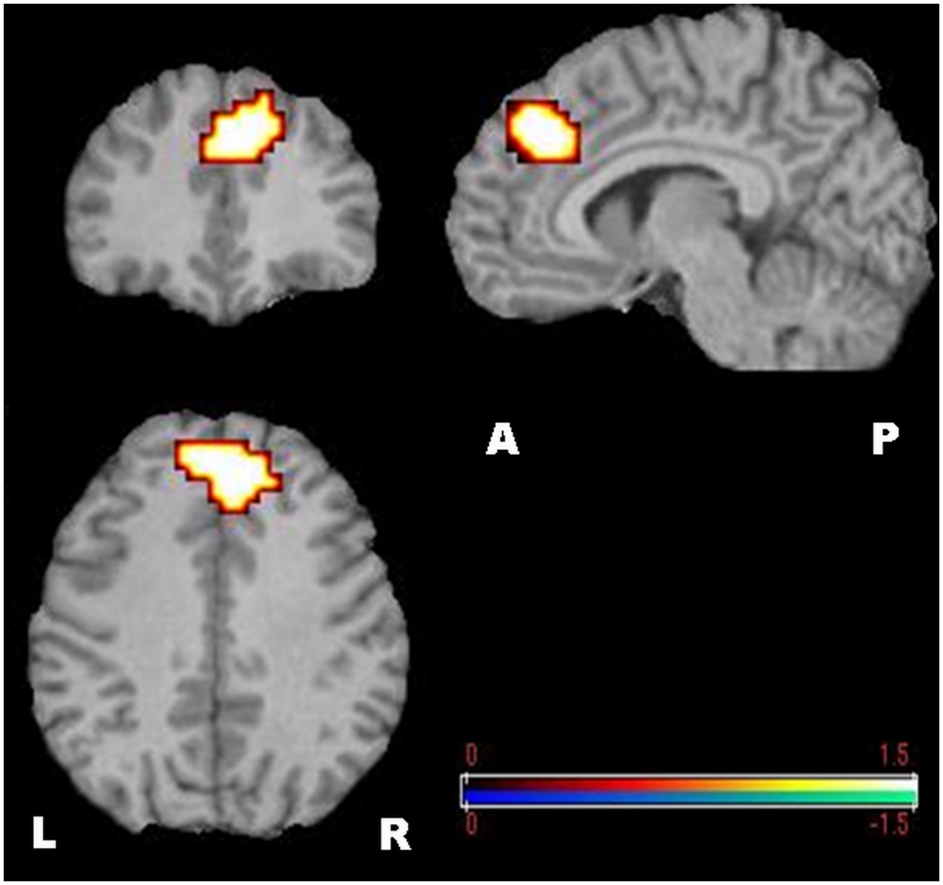
**SAM-permutation images of source power changes (event-related synchronization) in the theta (4–8 Hz) band**. Responses were calculated for the mental calculation (active state) vs. non-arithmetic condition (control state). The color bar represents pseudo-*t*-values. L, Left; R, Right; A, Anterior; P, Posterior.

The visual inspection of the MEG recordings revealed increased rhythmic theta activity at around 5–7 Hz when the subjects were engaged in mental calculation compared to the resting condition. This rhythmic theta activity appeared over the frontal regions bilaterally (Figure 2). Figure 3 shows the results of SAM analysis of the subjects (*n* = 8) with prominent theta waves in medial frontal areas during the arithmetic task compared to the resting state. In addition to midfrontal theta oscillations seen in all subjects, theta ERS was also seen in other cortical areas in some subjects, with inter-subject variability. These areas included the anterior temporal, orbitofrontal, and dorsolateral prefrontal cortex (Figure 3). The permutation test results indicated a significant increase in theta activity, or theta ERS, in the medial prefrontal cortex (BAs 8 and 9) and the adjacent dorsal part of the ACC (BA 32) during periods of focused attention on mental calculation (Figure 4). Table 1 summarizes the cortical distribution and values of task-induced activation or deactivation, as indicated by SAM-permutation analysis.

### Power changes in gamma frequency band

**Figure 5:**
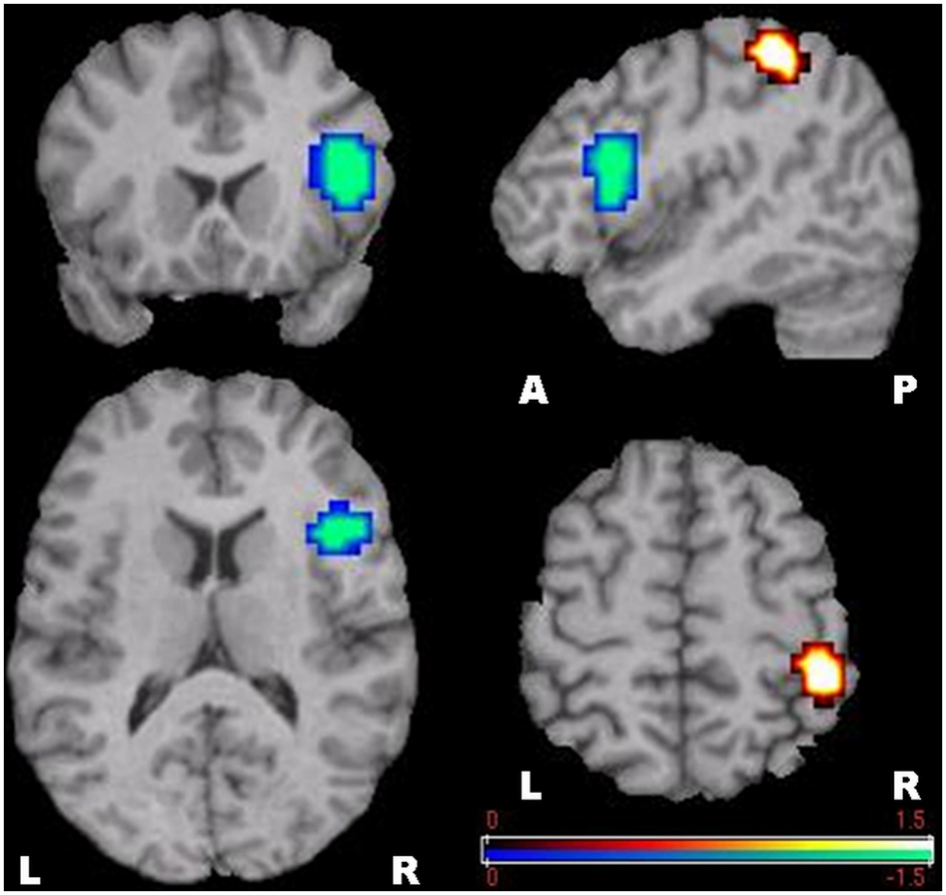
**SAM-permutation images of source power changes in the gamma (30–60 Hz) band**. Responses were calculated for the mental calculation (active state) vs. non-arithmetic condition (control state). Event-related synchronization was seen in the right posterior parietal cortex, and event-related desynchronization in the right inferior frontal gyrus. The color bar represents pseudo-*t*-values. L, Left; R, Right; A, Anterior; P, Posterior.

Focusing attention on mental calculation, specifically on serial arithmetic subtraction, resulted in significant source-power changes in gamma frequency bands over different cortical regions. There were no significant power changes in any of the other frequency bands. Time-frequency analysis for MEG channels in individual subjects showed a similar pattern of enhancement and suppression of oscillatory activity in the gamma band over parietal and frontal areas, respectively, predominantly in the right hemisphere. The statistical group analysis provided by SAM-permutation tests revealed that gamma activity (30–60 Hz) exhibited both significant ERS and ERD during the mental calculation periods. Gamma ERS was observed in the right intraparietal sulcus (IPS) and the adjacent posterosuperior and inferior parietal lobules, whereas the ERD was observed over the inferior frontal gyrus (BA 44) in the same hemisphere (Figure 5). Calculation-related gamma ERS and ERD values are provided in Table 1.

## Discussion

Using MEG and SAM-permutation analysis during continuous mental calculation, we clearly identified pronounced theta ERS, representing Fmθ, distributed over bilateral medial prefrontal regions and the dorsal area of the ACC (Figure 4). A striking finding was the identification of significant gamma power changes, in particular gamma ERS in the right IPS and adjacent cortex, and gamma ERD in the inferior frontal cortex that appeared concomitantly with Fmθ (Figure 5). This clearly shows that focusing attention on mental calculation results not only in Fmθ generation but also in the activation of neural networks involving the parietal and lateral prefrontal cortex, likely associated with the arithmetic processing of the task, with power changes in the gamma band representing the underlying neural activity.

### Frontal midline theta sources

Despite the body of information obtained from earlier EEG and MEG studies, to date, a precise delineation of brain structures involved in Fmθ generation has been difficult. This is due in part to a lower spatial resolution of EEG compared to MEG, the use of dipole modeling which can only detect center of gravity of activated regions rather than cortical volume of activation (Herdman et al., 2003), and the limitation of the lack of group statistical analysis in previous MEG-SAM studies (Ishii et al., 1999). In the present study, SAM-permutation analysis showed theta ERS specifically over the dorsal part of the ACC and adjacent medial prefrontal cortex bilaterally, when subjects were engaged in continuous mental calculation (Figure 4).

The ACC encompasses numerous specialized subdivisions that subserve a vast array of cognitive, emotional, executive, nociceptive and visuospatial functions (Bush et al., 2000; Womelsdorf et al., 2010; Ovaysikia et al., 2011). Interestingly, we noted that within the ACC, significant increase in theta power was observed particularly over the dorsal area (BA 32), which corresponds to the cognitive subdivision of the ACC. Among other functions such as control of motivation, error detection and working memory processing, the dorsal part of the ACC is implicated in modulation of attention, which is why this area is regarded as part of a distributed attentional network (Bush et al., 2000). Conflict resolution is another function traditionally associated with the ACC. In the context of arithmetic subtraction, this ACC function might be necessary to inhibit potential error responses during the task. Thus, our results are consistent with and extend the findings of previous investigations proposing the dorsal part or cognitive subdivision of the ACC and adjacent medial prefrontal cortex as the generators of Fmθ associated with focused attention and other cognitive functions during mental calculation (Ishihara and Yoshi, 1972; Sasaki et al., 1996; Asada et al., 1999; Ishii et al., 1999; Enriquez-Geppert et al., 2013).

It is noteworthy that enhanced theta activity is thought to reflect working memory processes, as well. For instance, frontal theta ERS has been associated with working memory load and attention demands in several neurophysiological studies (Gevins et al., 1997; Kahana et al., 2001; Jensen and Tesche, 2002; Onton et al., 2005). The simple subtraction task used in this study requires working memory processing, in particular the maintenance and manipulation of information, which is closely associated with medial prefrontal cortex (i.e., Brodmann areas 8 and 9) function. Taking this into account, we believe that, in addition to focused attention-related activation, the theta ERS observed in our study might also reflect working memory process associated with mental calculation.

In our study, three subjects out of eleven didn't show any prominent theta activity during the calculation task, even though they carried out the task like other subjects. There are several previous studies suggesting some influential parameters which could affect the appearance rate of Fmθ among individuals, such as anxiety level and personality traits (Inanaga, 1998). Although we didn't check the anxiety level and personality traits in this study, some possible behavioral, structural and genetic factors which might be associated with prominent theta activity can be investigated as our future application.

### Parietal cortex involvement in number processing

Little has been reported on gamma band activity and arithmetic processing in previous Fmθ experiments using mental calculation as attention-demanding task. Indeed, several EEG/MEG studies on Fmθ induced by arithmetic tasks focused exclusively on source localization of theta oscillations and did not analyze fast frequencies, including the gamma band (Iramina et al., 1996; Sasaki et al., 1996; Mizuhara et al., 2004; Missonnier et al., 2006; Doppelmayr et al., 2008). Consistent with our findings of pronounced gamma ERS in the right IPS (Figure 5), this cortical area has been a major site of activation in neuroimaging studies on number processing. Based on its systematic activation whenever numbers are manipulated, regardless of number notation, the IPS has been regarded as a potential substrate for quantity or numbers representation common to all arithmetic tasks (Rueckert et al., 1996; Simon et al., 2002; Dehaene et al., 2003, 2004; Kong et al., 2005). Of note, subtraction operation, which was used for mental arithmetic in this study, usually elicits greater IPS activation compared to other arithmetic operations, in particular multiplication and division. This is partly explained by the fact that multiplication tables, and even small addition facts, can be stored in rote verbal memory, hence placing minimal requirements on quantity manipulation, whereas subtraction problems are not learned by rote and therefore require genuine quantity manipulation (Simon et al., 2002; Dehaene et al., 2003).

We also noted significant gamma ERS in cortical areas adjacent to the IPS, namely the inferior and postero-superior parietal (PSP) lobules, which have also been implicated in numerical operations, including subtraction of two or more digits and counting (Dehaene et al., 2003). However, unlike the IPS, the PSP region is not specific to the number domain. Rather, it plays a central role in a variety of visuospatial tasks including orienting of attention, regardless of whether working memory process is involved or not (Mitchell and Cusack, 2008; Olson and Berryhill, 2009). This suggests that PSP cortex activation mainly reflects the processing of attended items. In this context it is interesting that the so called internal “number line,” a quasispatial representation on which numbers are organized by their proximity, can be likened to the core semantic representation of numerical quantity. Thus, it is conceivable that the same process of covert attention that operates to select locations in space can also be engaged when attending to specific quantities on the “number line” during mental calculation (Dehaene et al., 2003). This number-based spatial attention hypothesis supports the involvement of the PSP lobule not only in visuospatial processing, as previously reported, but also in non-visual mental arithmetic tasks, as suggested by our results.

### Gamma event-related synchronization (ERS) in cognitive processing

It is noteworthy that activation in areas subserving basic arithmetic processing and number-based spatial attention in the parietal lobe manifested specifically as an increase in power in the gamma band (Figure 5). This supports previous observations of sustained EEG gamma oscillations during high-level mental activities, such as reading, learning, emotion and arithmetic subtraction (Fitzgibbon et al., 2004; Luo et al., 2013). Our finding is consistent with evidence demonstrating that activation of cortical regions induced by cognitive processes generally translates into synchronization of rhythmic neural activity at frequencies above 40 Hz, the so-called gamma synchronization (Lachaux et al., 2008). Induced gamma response has also been reported to reflect activation of task modality-dependent networks or stimulus-related sensory/cognitive function in primary or association areas subserving the specific stimulus information processing (Jensen et al., 2007). Furthermore, synchronized gamma activity is considered to be involved in object representation, including internally driven representations (Bertrand and Tallon-Baudry, 2000) and in specific modalities of attention (Kaiser and Lutzenberger, 2003, 2005; Jensen et al., 2007). Taken together, our findings and those of earlier neuroimaging studies suggest that gamma band synchronization underlies number-related cognitive processing.

A separate line of research indicates that theta phase can modulate gamma power in certain brain regions (Scheffer-Teixeira et al., 2012). Kaplan et al. using MEG found that theta-gamma phase-amplitude coupling between medial prefrontal areas and medial temporal areas was linked to memory retrieval (Kaplan et al., 2014). Based on these findings, it would be interesting to explore in future studies whether theta and gamma phase-amplitude coupling between the medial prefrontal and parietal cortex, rather than activation of isolated cortical regions, might play a role in arithmetic processing.

### Gamma event-related desynchronization (ERD) in cognitive processing

In contrast to a gamma ERS phenomenon in the parietal cortex, as an index of stimulus-related local activation, the significant power changes in the inferior frontal gyrus consisted of gamma ERD (Figure 5). This speaks in favor of a functional disassociation between prefrontal and parietal cortices during arithmetic processing, as proposed by an fMRI study by Menon et al. (2000). Although there is no clear explanation for the existence of simultaneous gamma ERS and ERD during focused attention on mental calculation, this finding is in line with recent evidence indicating that performing attention-demanding cognitive tasks require not only activation of specific cortical regions but also deactivation of other regions that can interfere with the ongoing cognitive task, either in low-level sensory areas or high-level structures, such as the prefrontal cortex (Lachaux et al., 2008).

In this context, the gamma ERD found in the inferior frontal cortex may indicate that this region was not directly involved in the numerical processing. Rather, this region may play a more supportive role, for instance in managing parallel processes that might interfere with fast continuous subtractions, such as working memory-related processes or the speed of the calculation without seriously compromising arithmetic performance(Menon et al., 2000). Reports indicating that inhibitory control (inhibition of irrelevant information with working memory demands) is associated with a distributed network, involving the right dorsolateral prefrontal cortex (Garavan et al., 1999; MacDonald et al., 2000), ACC (Rubia et al., 2003), and the inferior parietal cortex (MacDonald et al., 2000; Garavan et al., 2002) provide further support to this argument.

In this study, we noted a right hemisphere laterality of the gamma source-power changes. Interestingly, previous fMRI and neuropsychological studies also found a predominant right hemisphere activation of frontal and parietal regions in association with mental subtraction (Fehr et al., 2007). Further, there is evidence that engagement in simple arithmetic, in particular subtraction operation, activates a neural network predominantly in the right hemisphere. This network is thought to serve as a common basis to which more regions in the left hemisphere are recruited for more difficult problems or different arithmetic operations (Kong et al., 2005).

Our findings should be interpreted with caution based on the limitation of the lack of behavioral performance data. This was due to the use of a mental arithmetic task to avoid movement-related artifacts during the MEG recordings. Previous neuroimaging studies using fMRI or EEG have also used this paradigm (Rueckert et al., 1996; Kawashima et al., 2004; Mizuhara et al., 2004). However, prior to experiments, the subjects in this study performed practice trials, during which answers were given orally and the accuracy of the calculations was checked. This allowed us to measure each subjects' arithmetic proficiency and to ensure that they were capable to perform well on the task. Despite the use of a purely mental task, we found cortical activation, as indicated by specific oscillatory power changes, in a frontal-parietal network thought to be involved in focused attention (Ishihara and Yoshi, 1972; Ishii et al., 1999) and numeric/quantity representations (Dehaene et al., 2003, 2004), concomitantly with Fmθ. This strongly suggests that the subjects were actually performing the arithmetic task during the recordings.

### Laterality of source-power changes

Consistent with results of earlier studies, we found that Fmθ appeared bilaterally over the medial prefrontal and ACC cortex. This confirms the involvement of both hemispheres in focused attentional processing. However, the gamma source-power changes in number processing areas and the prefrontal cortex, showed a unilateral distribution in the right hemisphere. This is unlikely to be related to the limitation of SAM beamformer of suppressing sources highly correlated in time across hemispheres to reduce noise in the signal (Brookes et al., 2007, 2008) because this effect generally applies to evoked-related responses which are time-locked to the onset of a specific stimulus in each single trial. Cognitive stimulus-induced oscillatory responses, however, jitter in the long range of time window after the stimulus onset from one trial to another (Pfurtscheller and Lopes da Silva, 1999; Tallon-Baudry and Bertrand, 1999). Thus, SAM is appropriate to reconstruct those task-related source-power changes in oscillatory activity or induced response, as it has been demonstrated in several MEG studies (Herdman et al., 2003; Gunji et al., 2007; Ishii et al., 2009).

A possible explanation for the right hemisphere laterality of the gamma source-power changes in this study may be based on the existence of sharp disassociations between arithmetic operations and calculation complexity (Menon et al., 2000; Gruber et al., 2001; Dehaene et al., 2003, 2004; Kong et al., 2005; Fehr et al., 2007; Ischebeck et al., 2009). As mentioned above, multiplication operations, which require arithmetic fact retrieval and rote memory, induce activation mainly of the left angular gyrus (Gruber et al., 2001; Dehaene et al., 2003, 2004; Ischebeck et al., 2009). This area which is associated with verbal number manipulation, appears to be also involved in complex arithmetic operations (Menon et al., 2000; Dehaene et al., 2003, 2004; Fehr et al., 2007; Grabner et al., 2007) along with the left inferior temporal (Gruber et al., 2001; Kong et al., 2005) and inferior parietal (Kong et al., 2005) cortex. Taking into account that we used simple subtraction as basic arithmetic operation, which is related to genuine quantity manipulations (Menon et al., 2000; Dehaene et al., 2003, 2004), it is not surprising that all those areas in the left hemisphere showing operation-specific activation were not found significantly activated in this study. Previous fMRI and neuropsychological reports suggesting a predominant right hemisphere activation of frontal and parietal regions in association with mental subtraction (Fehr et al., 2007) and with Arithmetical Reasoning Test performance (Langdon and Warrington, 1997) provide further support to our results.

Lesional studies have also provided evidence of right parietal cortex involvement in arithmetic processing. Dehaene and Cohen (1997) reported two acalculic patients who had structural lesions in the left subcortical areas or the right parietal cortex. They noted that the left lesional case had impaired rote arithmetic facts processing with preserved knowledge of numerical quantities. However, in line with our findings, the patient with right inferior parietal lesion showed a specific impairment of quantitative numerical knowledge, which was particularly remarkable for subtraction tasks (Dehaene and Cohen, 1997). Additionally, recent reports of intraoperative cortical electrostimulation in patients with brain tumors have confirmed an anatomofunctional organization for arithmetic processing within the right parietal cortex (Della Puppa et al., 2013).

Consistent with our SAM-permutation results, Micheloyannis et al. (2002) study using linear and non-linear EEG measures indicated that right hemisphere activation during simple arithmetic is manifested as increased power in the gamma band, while left or bilateral theta and alpha responses appear to be associated with the calculation strategy applied (De Smedt et al., 2009; Grabner and De Smedt, 2011). Further, there is evidence suggesting that engagement in simple arithmetic, in particular subtraction operation, activates a neural network predominantly in the right hemisphere, which serves as a common basis to which more regions in the left hemisphere are recruited for more difficult problems or different arithmetic operations (Kong et al., 2005). Based on this argument, we can speculate that the significant gamma synchronization seen in our study represent activation and connectivity of different brain areas, engaged in simple subtraction in the right hemisphere (i.e., right parietal cortex), and contralateral cortical areas could have been recruited if the complexity of the task was manipulated or other arithmetic operations were used. Although not directly tested in the current study, we suggest that MEG connectome might be able to provide novel insights on the biological mechanisms of higher cognitive functions and pathophysiology of neuropsychiatric diseases.

## Conclusion

The findings of this study should be interpreted in the context of brain activation associated particularly with focused attention on mental arithmetic subtraction. Using MEG and SAM-permutation analysis, our results confirm and extend those of previous EEG and MEG studies indicating that Fmθ is generated in medial prefrontal cortex and dorsal ACC during cognitive tasks requiring focused attention and working memory process. Moreover, our results suggest that right parietal cortical areas which subserve basic numerical processing and number-based spatial attention, namely the IPS and the adjacent postero-superior parietal lobule, respectively (Dehaene et al., 2003), are activated during mental subtraction operations. The main contribution of our work, however, is the identification of gamma ERS as the underlying neural activity of this parietal sources. In addition, gamma ERD occurred in the right lateral frontal cortex during performance of the mental task, likely representing a mechanism to interrupt transiently local neural communication in cortical regions not relevant to the ongoing cognitive task. Overall, our findings demonstrate the feasibility of using MEG and SAM-permutation analysis to determine cortical network of sources related to focused attention or arithmetic calculation, and their underlying neural activity.

### Conflict of interest statement

The authors declare that the research was conducted in the absence of any commercial or financial relationships that could be construed as a potential conflict of interest.
